# Design and methods of the ‘monitoring outcomes of psychiatric pharmacotherapy’ (MOPHAR) monitoring program – a study protocol

**DOI:** 10.1186/s12913-019-3951-2

**Published:** 2019-02-14

**Authors:** Mirjam Simoons, Henricus G. Ruhé, Eric N. van Roon, Robert A. Schoevers, Richard Bruggeman, Daniëlle C. Cath, Diny Muis, Johan Arends, Bennard Doornbos, Hans Mulder

**Affiliations:** 1 Department of Clinical Pharmacy, Wilhelmina Hospital Assen, Assen, The Netherlands; 2 Department of Psychiatry, Interdisciplinary Centre for Psychopathology and Emotion Regulation, University of Groningen, University Medical Centre Groningen, Groningen, The Netherlands; 3 Department of Pharmacy, Unit of Pharmacotherapy, -Epidemiology & -Economics, University of Groningen, Groningen, The Netherlands; 4 Department of Psychiatry, Radboudumc, Nijmegen, The Netherlands; 5 Department of Clinical Pharmacy and Clinical Pharmacology, Medical Centre Leeuwarden, PO Box 888, 8901 BR Leeuwarden, The Netherlands; 6 Rob Giel Research Centre, University of Groningen, University Medical Centre Groningen, University Centre for Psychiatry, Groningen, The Netherlands; 7 Present Address: Department of Specialized Training, Psychiatric Hospital MHS Drenthe, Outpatient Clinics, Assen, The Netherlands

**Keywords:** Physical health, Implementation, Somatic monitoring, Psychiatric pharmacotherapy, Outpatients, Health care organization

## Abstract

**Background:**

At many outpatient departments for psychiatry worldwide, standardized monitoring of the safety of prescribed psychotropic drugs is not routinely performed in daily clinical practice. Therefore it is unclear to which extent the drugs used by psychiatric outpatients are prescribed effectively and safely. These issues warrant structured monitoring of medication use, (pre-existing) co-morbidities, effectiveness and side effects during psychiatric outpatient treatment. Improvement of monitoring practices provides an opportunity to ensure that somatic complications and adverse drug effects are detected and dealt with in a timely manner. Structural support for data collection and follow-up tests seems essential for improvement of monitoring practices in psychiatric outpatients. The implementation of a structured somatic monitoring program as part of routine clinical practice, as we describe in this study protocol, may be a solution.

**Methods:**

In order to address these issues, we developed the innovative program ‘Monitoring Outcomes of Psychiatric Pharmacotherapy (MOPHAR)’. MOPHAR is an infrastructure for implementation of standardized routine outcome monitoring (ROM; including standardized monitoring of treatment effect), monitoring of adverse psychotropic medication effects in psychiatric outpatients, encompassing both somatic adverse effects (e.g. metabolic disturbances) and subjective adverse effects (e.g. sedation or sexual side effects) and medication reconciliation.

**Discussion:**

In the MOPHAR monitoring program, a nurse performs general and psychotropic drug-specific somatic screenings and provides the treating mental health care providers with more and better information on somatic monitoring for treatment decisions. Given our experience regarding implementation of the MOPHAR program, we expect that the MOPHAR program is feasible and beneficial for patients in any MHS organisation. This paper describes the objectives, target population, setting and the composition and roles of the treatment team. It also indicates what measurements are performed at which time points during outpatient treatment in the MOPHAR monitoring program, as well as the research aspects of this project.

**Trial registration:**

MOPHAR research has been prospectively registered with the Netherlands Trial Register on 19th of November 2014. (NL4779).

**Electronic supplementary material:**

The online version of this article (10.1186/s12913-019-3951-2) contains supplementary material, which is available to authorized users.

## Background

Severe mental illness patients are expected to live a 13–30 year shorter life compared to the general population [1, 2]. Somatic co-morbidities such as cardiovascular disease, nutritional and metabolic diseases and pain may account for approximately two-thirds of this excess mortality [1, 3, 4]. This increased somatic morbidity and mortality may be contributed to by various factors, including an unhealthy lifestyle (directly or indirectly associated with psychopathology of the patient) and disparities in health care provision and access, associated with the psychiatric disease [1, 5]. Also, psychotropic drugs may cause and/or increase the vulnerability of patients with mental illness to somatic adverse drug effects [1, 6]. Metabolic syndrome and other somatic complications do not only occur in schizophrenia patients or patients using antipsychotics. Mood disorders are also known to negatively influence lifestyle [7, 8]. Moreover, these disorders are commonly treated with combinations of lithium, mood stabilizers, antipsychotics and antidepressants. These patients are therefore at risk for developing somatic complications too [9, 10]. In addition, psychiatric patients are generally less inclined to use health care services and have a decreased perception of illness compared to the general population [11].

Worldwide, at many specialized outpatient clinics for psychiatric disorders, systematic monitoring of the safety of prescribed drugs is not routinely performed in daily clinical practice. Previous research from our group has indicated that medication reconciliation and monitoring of somatic parameters are not routine clinical practice at outpatient departments for mood and anxiety disorders in the north of The Netherlands [12, 13]. Likewise, in a large benchmarking audit in lithium-treated patients from The United Kingdom, weight, body mass index (BMI) and waist circumference had not been recorded in 72% of 2976 patients, (follow-up) tests on kidney and thyroid function had not been performed in 19 and 18% of patients respectively, and lithium serum concentration had not been taken in 9% [14]. A meta-analysis of 39 studies (*n* = 218,940) investigating metabolic syndrome in patients with mostly schizophrenia or related disorders and that were using antipsychotics, showed that baseline metabolic screening before commencement of pharmacotherapy was suboptimal and in more than 50% of patients only blood pressure and triglycerides blood concentrations were checked [15]. Research in somatic departments shows that between 90 and 100% of HIV patients are regularly screened on hypertension, diabetes and dyslipidaemia, which is considerably higher than 40–70% in psychiatric outpatients taking antipsychotics. This suggests particularly poor monitoring in patients with psychiatric problems [16]. Furthermore, there may be considerable medication discrepancies between the medication overview at the psychiatric outpatient clinic and the actual medication use by the patient [12]. In conclusion, monitoring of side effects (associated with prescribing psychotropic medication) and medication use has generally not been systematically implemented in daily psychiatric practice. Therefore it is unclear to which extent the drugs used by psychiatric outpatients are prescribed safely. These issues warrant systematic somatic monitoring of (pre-existing) co-morbidities, side effects and medication use, during psychiatric outpatient treatment.

Improving monitoring practices may ensure that somatic co-morbidities and adverse drug reactions are detected and treated in a timely manner. Unfortunately, the introduction of new guidelines, education materials, (national) quality improvement programs or consensus statements alone have shown only minimal improvement in monitoring practices [15, 17–20]. There are several potential reasons for this lack of effectiveness of these strategies. For example, the documents or materials may not reach all relevant health care professionals in the expected time frame, awareness of the need for improvement of monitoring practices may be lacking or resources for local implementation of the recommendations may be unavailable.

Taken together, structural support of mental health care professionals for data collection and follow-up testing at a local or regional level seems essential for improvement of monitoring practices in psychiatric outpatients. Implementing a structured somatic monitoring program that is incorporated in routine clinical practice may be a solution [21]. In order to address these issues, we developed the innovative monitoring program ‘Monitoring Outcomes of Psychiatric Pharmacotherapy (MOPHAR)’. MOPHAR is an infrastructure for implementation of standardised routine outcome monitoring (ROM; including standardised monitoring of treatment effect), monitoring of objective somatic adverse effects including metabolic disturbances as well as subjective symptoms such as sedation or sexual side effects of psychiatric pharmacotherapy and medication reconciliation in outpatients. This monitoring program added to standard psychiatric treatments, also provides the opportunity to build a patient registry for the conduct of research on topics such as physical complications, side effects of medication and monitoring care in psychiatric outpatients.

Although it seems logical to monitor and treat known complications of psychiatric disease and psychotropic medication, the evidence for benefits of monitoring specific (sets of) parameters (and subsequent treatment if indicated) in terms of for example less somatic complications, better quality of life or shorter treatment duration is lacking. The (cost)effectiveness of monitoring of specific (sets of) parameters needs to be established to provide an evidence base for investing in implementation of systematic monitoring programs and reimbursement by health care insurance companies. This paper describes the research aspects of the MOPHAR study as well as the process and measurements of the MOPHAR monitoring program.

## Methods/design

### MOPHAR research

Apart from a somatic monitoring care path for routine clinical practice, the MOPHAR monitoring program also provides the opportunity for long-term (longitudinal) prospective and retrospective observational cohort studies. The large amount of information collected in the patient registry of MOPHAR can be used for research: many questions may be answered in retrospective studies, including association studies and prediction models on the effect and side effects of psychotropic drugs. Because apart from all current patients, also all new patients are asked for informed consent to be included in MOPHAR, the sample size will increase in time. The sample sizes for analyses will be pre-specified per individual research question.

Next to retrospective studies, MOPHAR also gives the perspective for future prospective studies. After implementation of MOPHAR there is a structured program in place with uniform moments for evaluation by a MOPHAR nurse. These moments can be used for future prospective interventions as well. Furthermore, patient and treatment characteristics are gathered systematically thereby allowing selection of patients suited for specific prospective studies.

The general research objectives are:

1. To investigate the association between patient characteristics and outcomes (e.g. (cost)effectiveness, adverse effects) of psychiatric pharmacotherapy. Amongst others the association between pharmacogenetic determinants/biomarkers and the prevalence of adverse effects of antidepressants will be investigated.
2. To investigate the association between the use of specific psychotropic drugs and adverse effects like metabolic abnormalities in selected samples and the unselected population (population-based research). In addition we will be able to set up intervention studies targeting such adverse effects.

Our research objectives reflect both the aim to investigate how we can most efficiently detect the relevant signals for somatic complications and the aim to investigate how we can predict which patients will probably benefit from specific psychotropic drugs and/or are vulnerable for specific side effects. Results of these studies can be used to prevent, monitor and treat adverse effects in the near future. More specific research questions will be formulated per individual study within MOPHAR, along with theoretical frameworks and specific definitions, if applicable. In addition, the statistical analyses plans will be described per individual research question. We expect to use for example regression analysis techniques and longitudinal data analysis techniques (for clustered data), including mixed effects models.

#### Informed consent

MOPHAR research has been registered with the Netherlands Trial Register (NL4779; https://www.trialregister.nl/trial/4779). The research aspects of MOPHAR were approved by the independent medical ethics committee (RTPO 928, rTPO Leeuwarden, The Netherlands), and all participants provide written informed consent. We ask general informed consent to conduct research on the data collected in the MOPHAR monitoring program including the linkage of the clinical data with an extra blood sample obtained for MOPHAR research (see below under ‘extra blood sample’). Subjects can withdraw from further participation in the MOPHAR research cohort at any time for any reason without any consequences regarding their treatment and MOPHAR monitoring care.

#### Study population

For every research question addressed in MOPHAR, the appropriate study population will be determined within the MOPHAR cohort from the patient registry. In general, eligible patients meet the following inclusion criteria: older than 18 years of age and visiting an outpatient department of MHS Drenthe (first time or follow-up visit, i.e. newly referred and current patients) are eligible for inclusion in MOPHAR).There are no general exclusion criteria for inclusion in the MOPHAR patient registry.

#### Extra blood sample

For research purposes, an extra blood sample (20 ml) will be taken from each subject. This blood sample will be taken at the same time as one of the blood sample withdrawals for routine clinical practice. Therefore, no additional venepuncture is necessary and no additional risks are associated with this single study procedure. This blood sample can be used for future research (for example, pharmacogenetics and biomarker research) to investigate associations between drug or patient characteristics and treatment success and/or the prevalence of somatic side effects concerning scientific questions related to psychiatric health issues for which the patient visited the outpatient department.

### Objectives of the MOPHAR monitoring program

The primary objective of the MOPHAR monitoring program is to systematically provide mental health care providers with more and better information for treatment decisions and to facilitate monitoring of the treatment effect and adverse effects of psychiatric pharmacotherapy in psychiatric outpatients.

Secondary objective is to enable routine collection of longitudinal monitoring data of daily psychiatric practice for research purposes. The general research objectives have been discussed above.

### Target population and setting

The MOPHAR monitoring program targets adult patients (≥ 18 years) referred to mental health care outpatient clinics for any psychiatric diagnosis. Patients can be referred by their general practitioner or by a mental health care treatment officer from another department or institution. Any information on the psychiatric symptoms, co-morbidities and medication use that has been noted in the referral letter is incorporated in the available information for the initial visit for MOPHAR. After referral and initial visit at the outpatient clinic, regular mental health care at the general practitioner’s practice stops. MOPHAR accommodates patients either at initial visit or already in treatment at the outpatient clinic at the time of implementation.

The MOPHAR monitoring program is currently implemented at a large secondary community mental health care outpatient department. However, in its current form, it can be implemented at any mental health care outpatient clinic serving a broad population of persons with a (severe) mental illness. While a core set of elements and monitoring measurements is provided in MOPHAR, the current program as described in this paper does not preclude access to other somatic services or program amendments fitted to specific populations (e.g. disease-specific measurements or questionnaires or paper-based instead of online questionnaires for elderly patients).

### The MOPHAR treatment team

The MOPHAR treatment team is multidisciplinary. The MOPHAR team comprises the regular treatment team with at least one psychiatrist, at least one psychiatric nurse trained in the somatic screening of MOPHAR and a secretary. However, usually more than one person per discipline is involved, as well as a psychologist and a nursing specialist. There is a flexibility in the size and composition of the team.

The roles of the different team members can be described as follows. The secretary plans the appointments and invites the patient, which marks the start of the MOPHAR monitoring program for individual patients. The psychiatric nurse performs the MOPHAR screenings. To this end, in a one-day session the psychiatric nurses are trained by the MOPHAR pharmacist in the logistics of the MOPHAR monitoring program and how to perform medication reconciliation, how to enter the medication use in the electronic prescribing system and how to register the MOPHAR screening results. The medication prescriber (i.e. psychiatrist or nursing specialist) is responsible for decisions on and execution of interventions and follow-up based on the results of the MOPHAR screenings along with the psychiatric treatment. The team must identify a clear workflow regarding the communication of results with other relevant health care professionals (e.g. general practitioner). At MHS Drenthe, the primary treatment officer sends a summary of the findings from the MOPHAR screenings to the general practitioner. The prescriptions are sent to the community pharmacy.

Members of the MOPHAR treatment team may have collateral responsibilities to MOPHAR patients or other non-MOPHAR (inpatient) teams. In addition, the nurses can be scheduled interchangeably for different outpatient teams to perform MOPHAR screenings if necessary. This flexibility may be a major appeal of the MOPHAR model of somatic monitoring for mental health care institutions. Since somatic monitoring is in part recommended in Dutch guidelines, MOPHAR is meant to be fitted into the usual workflow of the team. However, extra resources are needed for training, supervision, implementation and support. Implementation and project support are provided by a project manager and a pharmacist to ensure project progress and resolve practical issues. The pharmacist is responsible for training the MOPHAR nurses (e.g. medication reconciliation) and the quality assurance of the established (psychotropic drug-specific) monitoring protocols. In addition, the pharmacist can be consulted by the treatment team with medication-related questions.

### The MOPHAR monitoring process and protocols

**Fig. 1:**
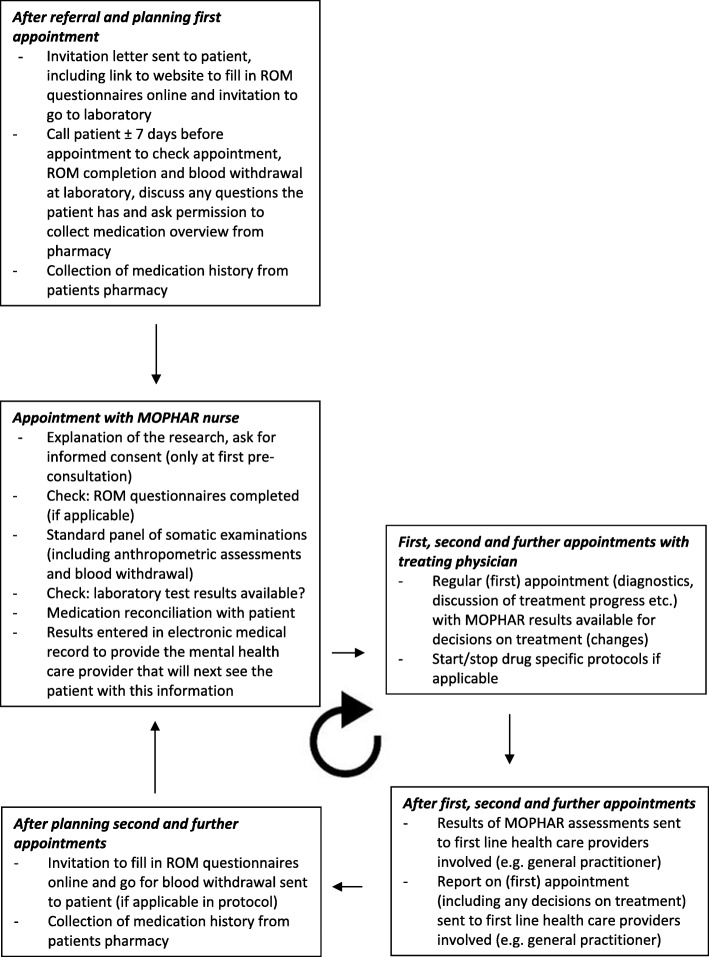
Schematic presentation of health care and study procedures in MOPHAR. All procedures shown are performed as a part of routine daily clinical practice. Routine Outcome Monitoring (ROM; online patient-filled questionnaires) has to be completed at certain time points, but not with all MOPHAR appointments

Figure 1 shows the general process of the MOPHAR monitoring program. MOPHAR is an addition to the established routine clinical practice at the outpatient clinics. Because outpatients are simultaneously treated for their psychiatric disorder by different mental health care providers (e.g. a psychiatrist, psychologist, nursing specialist (nurse with a Master of Advanced Nursing Practice, allowed to treat patients independently or in some specialized settings under supervision of the psychiatrist) and/or psychiatric nurse), the appointments for a MOPHAR screening and the invitations to fill in online questionnaires are planned together as much as possible, shortly before the appointments with the mental health care provider(s). Frequency of attendance ranges between once in 3 months and twice weekly, depending on factors such as stage and acuteness of illness, illness severity and intensity of treatment; for MOPHAR they visit the clinic at least at initial visit and yearly, with additional (combined) visits if they (start to) use psychotropic medication.

#### Somatic screening at first appointment

A general somatic screening is performed at the first appointment, irrespective of (differential) diagnosis or medication use. This general somatic screening (±45 min) serves to screen for existing somatic co-morbidities, side effects of drugs already in use (e.g. metabolic disturbances), and potential (additional) causes of the mental illness (e.g. thyroid dysfunction for depression). In addition, it may serve as a baseline screening before start of psychotropic drug treatment if applicable.

#### Online patient-filled questionnaires

In the invitation letter for the first appointment, patients are asked to fill in questionnaires about their demographics, family history of psychiatric disease, smoking, alcohol and illicit substance use and previous psychiatric (pharmacotherapeutic) treatments. These questionnaires have been developed by the department of Psychiatry of the University Medical Centre Groningen, The Netherlands (HGR) [22]. In addition, the World Health Organisation Disability Assessment Schedule (WHO-DAS) 2.0 [23] and the Diagnostic and Statistical Manual of Mental Disorders (DSM)-5-level 1-questionnaire [24] on general psychiatric symptoms are filled in. The WHO-DAS 2.0 is a generic assessment of a patient’s health and disability, and with the results of the DSM-5-level 1-questionnaire the psychiatrist can assess in which mental health domains a patients experiences symptoms that need further examination because they may have a significant influence on the treatment and prognosis. The Outcome Questionnaire (OQ)-45 is added for non-elderly adult patients, to monitor treatment outcomes and general functioning [25]. Furthermore, an 18-item questionnaire is filled in about the presence of subjective symptoms and potential drug side effects. This questionnaire, called the Somatic Mini Screen (SMS), is developed by MHS Central, The Netherlands, and is in the process of validation (internal validity has been confirmed, inter- and intra-rater reliability is currently investigated). Lastly, at least one disorder-specific questionnaire is added to the set, depending on the patient population (e.g. the patient-completed self-report of the Inventory of Depressive Symptomatology (IDS-SR) in mood disorder patients). All questionnaires can be filled in by the patient through an online secured patient portal, that is integrated with the electronic medical records. This takes patients on average 60–90 min in total. We currently use RoQua, which is a patient portal that is accessible via the electronic medical records and is used by several associated mental health care institutions [26]. In case a patient does not have access to or skills in using a computer and/or internet or the electronic medical records are unavailable, a paper based version of the questionnaires can be completed. Eventually, the whole MOPHAR program can be implemented non-electronically. Furthermore, and alternatively, extra computers are available at the outpatient services that patients can use to fill in the questionnaires, sometimes with the aid of the nurse. The nurse checks whether all requested questionnaires have been filled in completely before the screening, so any missing information can be added during the appointment.

#### Screening appointment with MOPHAR nurse

During the MOPHAR screening visit, roughly four types of monitoring information are collected by the trained nurse (Fig. 1). First, a basic physical examination, including measurements of body mass index (BMI), waist circumference, blood pressure and heart rate. An electrocardiogram (ECG) can be added on indication.

Second, laboratory measurements. A nursing specialist or doctor orders the laboratory tests on a paper-based laboratory order form. The laboratory associated with MOPHAR has developed a dedicated laboratory order form for MOPHAR, on which the applicant can order all test from one measurement moment with one check. The nurse can perform the venepuncture, but patients from most teams are asked in the invitation for the appointment to go to the laboratory for blood withdrawal in the week before the screening with the laboratory order form sent along with the invitation. The total set of physical and laboratory measurements collected is shown in Supplemental Table 1 [see Additional file 1]. This protocol has been written in 2014 by a Dutch multidisciplinary working group, consisting of psychiatrists (including BD and HGR), (hospital) pharmacists (HM, MS) and a clinical chemist. The monitoring recommendations were based on the available national and international relevant monitoring guidelines to start with [27–31], but since there was a paucity thereof, the protocol was mostly based on clinical experience and expert opinion of the members of the working group. In 2015, the new guideline ‘Somatic screening of patients with a severe mental illness’ was published in The Netherlands, in which similar recommendations were described to those in the MOPHAR protocols [32].

Third, two structured interviews: one regarding somatic disease history of the patient and first-degree family members and the other regarding the patient’s lifestyle, including physical exercise and diet. The nurse completes these with the patient through the online portal in the electronic medical records.

Last, medication reconciliation. This is performed by the nurse through a combination of the pharmacy records and patient counselling. In preparation of the screening appointment, the nurse requests a medication overview (including medication and allergies or intolerances) from the community pharmacy of the patient by fax. At the screening appointment, the nurse reconciles this overview with the actual medication use by interviewing the patient. Medication reconciliation provides an up-to-date and complete medication overview including all drugs currently in use and all medication allergies or intolerances that the nurse enters in the electronic prescribing system. In case of relevant medication discrepancies (compared to the pharmacy records), the MOPHAR nurse will notify the psychiatrist/nursing specialist.

#### Availability of the screening results

The information collected via questionnaires beforehand and during the MOPHAR screening is immediately available to the mental health care provider via the patient portal and serves as a starting point for the anamnesis, psychiatric examination and a (semi-)structured interview for diagnostic purposes. The patient portal generates a summary of all information collected at the MOPHAR appointment. This summary selects a set of pre-specified most relevant parameters for a quick assessment of the clinical status of the patient, together with the information on medication use and laboratory tests.

#### Yearly somatic screenings

The general somatic screening at the first appointment is repeated yearly in all patients (±30 min), irrespective of psychiatric diagnosis or medication use. However, with respect to the patient-filled online questionnaires, only the smoking/alcohol/illicit substance use questionnaire, the WHO-DAS 2.0, the DSM-5 screener, and the SMS are repeated at the yearly screening as well as the disorder-specific questionnaire and OQ45 (if applicable).

#### Psychotropic drug-specific monitoring

In addition to the general somatic screenings at the first appointment and yearly thereafter, the MOPHAR nurse conducts additional screenings according to drug-specific monitoring protocols if a patient starts with or already uses one or more psychotropic drugs (±30 min per appointment). To this end, the abovementioned multidisciplinary working group has additionally written MOPHAR monitoring protocols per psychotropic drug (class). The monitoring protocols are shown in Additional files 2, 3, 4, 5, 6, 7, 8, 9, 10, 11, and 12 [27, 29, 30, 33–38]. The time points for the follow-up measurements differ per drug because of the different timeline of occurrence of side effects, but have been clustered as much as possible within each drug and between drugs to reduce the number of appointments and venepunctures. This makes the protocols uniform and enables clustering of follow-up measurements in patients using multiple psychotropic drugs.

In order to monitor subjective side effects, the SMS questionnaire is repeated three monthly when psychotropic medication is used. The physical exercise and lifestyle questionnaire (filled in by the nurse during a MOPHAR appointment) may also be repeated in the course of monitoring of psychotropic medication use.

Medication reconciliation is performed by the nurse at MOPHAR appointments or by the medication prescriber (i.e. psychiatrist or nursing specialist) if medication is prescribed, stopped or changed.

### Interpretation and follow-up of MOPHAR results

The summary generated from the patient portal, the medication overview from the electronic prescribing system and the laboratory test results together provide a full picture of the patient for the weekly to monthly multidisciplinary meeting where interventions and follow-up are planned [39]. A recent study by Bruins et al. (2016) showed that despite prevalences of the metabolic syndrome in > 50% at three yearly assessments in the PHAMOUS monitoring program for schizophrenia patients, half of the patients were not treated for their metabolic risk factors [39]. We will propose standardized interventions to facilitate the treatment of and follow-up on deviating test results by the responsible health care provider.

### Protocol evaluation

Apart from the abovementioned adjustments to fit specific populations, the core set of monitoring program elements will be adjusted over time. There is on ongoing debate regarding the necessity and appropriate frequency of monitoring of parameters such as the ECG [40, 41], liver function [42] and blood counts [43]. Also, monitoring items might be added to the program. For example, pharmacogenetics testing is not part of the protocol but the multidisciplinary group might decide to add pharmacogenetics testing to the program in the future [44]. The protocol therefore needs a yearly evaluation in a plan-do-check-act cycle to keep it up-to-date and adjusted to best clinical practices and new guidelines.

## Discussion

### MOPHAR current status and future perspectives

The MOPHAR monitoring program is currently incorporated in routine psychiatric care at the outpatient departments of MHS Drenthe after the assignment and approval for the implementation from the general board of MHS Drenthe. Eventually, all approximately 5700 adult patients with a (differential) diagnosis of a psychiatric disorder who are annually referred to a psychiatrist or psychologist at the MHS Drenthe outpatient departments will be asked to participate in MOPHAR. After that, we aim to implement MOPHAR at other mental health care institutions and also include patients from these other centres in MOPHAR research.

To the best of our knowledge, we are the first to describe a comprehensive monitoring program that actively supports mental health care professionals to implement guideline-concordant general somatic and psychotropic-specific monitoring of psychiatric patients in daily clinical practice, that is flexible to accommodate patients with any psychiatric diagnosis. Two monitoring programs have been described in the literature which, although with a similar level of active support, focused on psychotic patients only and/or on metabolic syndrome screening and monitoring [45, 46].

Implementation of MOPHAR started at the outpatient department for bipolar disorders. First appointment somatic screenings took place from January 2016 onward and yearly somatic screenings from November 2016 to synchronize MOPHAR with the individual yearly treatment evaluation schemes for patients that were already in treatment. Results of the first appointment somatic screenings for the outpatient department for bipolar disorders have been reported separately [47].

At this moment a general practitioner is not part of the treatment team. In the near future we would like to add this professional in order to ensure patient-centred care, including the treatment of detected somatic complications and adverse drug effects. Other potential future innovations of the program are a digital assurance system to ascertain protocol adherence and standardized interventions on aberrant test results where possible. In addition, the monitoring program may be adjusted for implementation in first-line health care-organizations, thereby serving the target population throughout the continuum of relevant (mental) health care providers for psychiatric outpatients.

## Conclusion

Psychiatric patients are vulnerable for somatic co-morbidities and side effects of psychotropic medication. However, current monitoring frequencies of somatic health of these patients may be low. There is a need for structural support for improvement of somatic monitoring practices in psychiatric outpatients in line with available monitoring guidelines. The active implementation of a structured monitoring program in which somatic monitoring is ensured as part of routine clinical will provide be a possible solution. In addition, it provides the opportunity to establish a patient registry for research purposes. In the MOPHAR monitoring program, a nurse performs general and psychotropic drug-specific somatic screenings and provides the treating mental health care providers with more and better information on somatic monitoring for treatment decisions. Given our experience regarding implementation of the MOPHAR program, we expect that the MOPHAR program including the research aspects is feasible and beneficial for patients in any MHS organisation.

## Additional files

Additional file 1: **Table S1.** MOPHAR protocol for baseline/yearly screening (DOCX 19 kb)

Additional file 2: **Table S2.** MOPHAR monitoring protocol tricyclic antidepressants (TCAs) (DOCX 19 kb)

Additional file 3: **Table S3.** MOPHAR monitoring protocol selective serotonin reuptake inhibitors (SSRIs) (DOCX 18 kb)

Additional file 4: **Table S4.** MOPHAR monitoring protocol selective serotonin and noradrenaline reuptake inhibitors (SNRIs) (DOCX 18 kb)

Additional file 5: **Table S5.** MOPHAR monitoring protocol Monoamide oxidase inhibitors (MAOIs) (DOCX 18 kb)

Additional file 6: **Table S3.** MOPHAR monitoring protocol other antidepressants (trazodone, mianserin, mirtazapine, bupropion, vortioxetine, agomelatine, hypericum) (DOCX 18 kb)

Additional file 7: **Table S7.** MOPHAR monitoring protocol antipsychotics other than clozapine (DOCX 18 kb)

Additional file 8: **Table S8.** MOPHAR monitoring protocol clozapine (DOCX 18 kb)

Additional file 9: **Table S9.** MOPHAR monitoring protocol lithium (DOCX 29 kb)

Additional file 10: **Table S10.** MOPHAR monitoring protocol carbamazepine (DOCX 19 kb)

Additional file 11: **Table S11.** MOPHAR monitoring protocol valproic acid (DOCX 17 kb)

Additional file 12: **Table S12.** MOPHAR monitoring protocol lamotrigine (DOCX 17 kb)

## Abbreviations

BMI: Body Mass Index
DSM: Diagnostic and Statistical Manual of Mental Disorders
ECG: Electrocardiogram
IDS-SR: Inventory of Depressive Symptomatology-Self Report
MHS: Mental Health Services
MOPHAR: Monitoring Outcomes of Psychiatric Pharmacotherapy
NTR: Netherlands Trial Register
OQ: Outcome Questionnaire
ROM: Routine Outcome Monitoring
rTPO: regionale Toetsingscommissie Patiëntgebonden Onderzoek (in Dutch; medical ethics committee)
SMS: Somatic Mini Screen
WHO-DAS: World Health Organisation Disability Assessment Schedule
