# Supplementary material for: Design and methods of the ‘monitoring outcomes of psychiatric pharmacotherapy’ (MOPHAR) monitoring program – a study protocol
Source: BMC Health Serv Res. 2019 Feb 14;19:125. doi: 10.1186/s12913-019-3951-2 (PMC6376699; doi:10.1186/s12913-019-3951-2)
Supplement: Supplementary file 1 — Table S1. MOPHAR protocol for baseline/yearly screening (DOCX 19 kb) [file 12913_2019_3951_MOESM1_ESM.docx]

**Supplemental table 1. MOPHAR protocol for baseline/yearly screening**

| *Anthropometrics* | |
| --- | --- |
| Length | X |
| Body weight | X |
| BMI | X |
| Waist circumference | X |
| *Cardiovascular measurements* | |
| Blood pressure (sitting/supine/standing) | X |
| Heart rate | X |
| Electrocardiogram | X^1^ |
| *Blood cells* | |
| Hemoglobin | X |
| Hematocrit | X |
| Leucocytes + differential | X^2^ |
| Thrombocytes | X |
| *Electrolytes* | |
| Sodium | X |
| Potassium | X |
| Calcium | X |
| *Kidney function* | |
| Creatinin | X |
| Estimated Glomerular Filtration Rate (eGFR) | X |
| *Liver function* | |
| Alkaline phosphatase | X |
| Alanine transaminase | X |
| Gamma-glutamyltransferase | X |
| *Thyroid function* | |
| Thyroid-stimulating hormone + free thyroxine 4 (FT4) | X^3^ |
| *Blood lipids* | |
| Triglycerides (fasting) | X |
| Cholesterol | X |
| Low Density Lipoprotein | X |
| High Density Lipoprotein | X |
| *Glucose* | |
| Fasting glucose | X^4^ |
| *Other measurements* | |
| Albumin | X |
| Vitamin B12 | X^5^ |
| Folic acid | X^5^ |
| Prolactin | X^6^ |
| Temperature | X |
| Pregnancy test | X^7^ |

^1^ With cardiac anamnesis, age >60 years of use of one or more QTc-prolonging drugs

^2^ Differential only in case of a deviating leucocyte count

^3^ FT4 only in case of a deviating thyroid-stimulating hormone level

^4^ HbA_1C_ (combined with a non-fasting glucose) in case a fasting glucose cannot be determined

^5^ On indication, in any case with age >65 years

^6^ On indication, in any case with young adults and for example in case of congenital or historic prolactin level deviations

^7^ In case of uncertainty about a potential pregnancy with women of child-bearing age
