# Supplementary material for: Design and methods of the ‘monitoring outcomes of psychiatric pharmacotherapy’ (MOPHAR) monitoring program – a study protocol
Source: BMC Health Serv Res. 2019 Feb 14;19:125. doi: 10.1186/s12913-019-3951-2 (PMC6376699; doi:10.1186/s12913-019-3951-2)
Supplement: Supplementary file 8 — Table S8. MOPHAR monitoring protocol clozapine (DOCX 18 kb) [file 12913_2019_3951_MOESM8_ESM.docx]

**Supplemental table 8. MOPHAR monitoring protocol clozapine**

|  | T = 0 | During dose adjustment | At least one measurement between T=3 weeks and T=2 months | T = 3 months | T = 6 months | Every 3 months | Yearly | On indication |
| --- | --- | --- | --- | --- | --- | --- | --- | --- |
| *Anthropometrics* | | | | | | | | |
| Length | X |  |  |  |  |  |  | X |
| Body weight | X |  | X | X | X |  |  | X |
| BMI | X |  | X | X | X |  |  | X |
| Waist circumference | X |  | X | X | X |  |  | X |
| *Cardiovascular measurements* | | | | | | | | |
| Blood pressure (sitting/supine/standing) | X |  | X | X | X |  |  | X |
| Heart rate | X |  | X | X | X |  |  | X |
| Electrocardiogram | X^1^ |  | X^1^ |  |  |  |  | X^2^ |
| *Blood cells* | | | | | | | | |
| Hemoglobin | X |  |  |  |  |  |  |  |
| Hematocrit | X |  |  |  |  |  |  |  |
| Leucocytes | X | X^3^ | | | | | | X |
| Differential | X | X^3^ | | | | | | X |
| Thrombocytes | X |  |  |  |  |  |  |  |
| *Electrolytes* | | | | | | | | |
| Sodium | X |  |  |  |  |  |  |  |
| Potassium | X |  |  |  |  |  |  |  |
| Calcium | X |  |  |  |  |  |  |  |
| *Kidney function* | | | | | | | | |
| Creatinin | X |  |  |  |  |  |  |  |
| Estimated Glomerular Filtration Rate (eGFR) | X |  |  |  |  |  |  |  |
| *Liver function* | | | | | | | | |
| Alkaline phosphatase | X |  |  |  |  |  | X^4^ | X |
| Alanine transaminase | X |  |  |  |  |  | X^4^ | X |
| Gamma-glutamyltransferase | X |  |  |  |  |  | X^4^ | X |
| *Thyroid function* | | | | | | | | |
| Thyroid-stimulating hormone + free thyroxine 4 (FT4)^5^ | X |  |  |  |  |  |  |  |
| *Blood lipids* | | | | | | | | |
| Triglycerides (fasting) | X |  |  | X | X |  | X | X |
| Cholesterol | X |  |  | X | X |  | X | X |
| Low Density Lipoprotein | X |  |  | X | X |  | X | X |
| High Density Lipoprotein | X |  |  | X | X |  | X | X |
| *Glucose* | | | | | | | | |
| Fasting glucose^6^ | X |  |  | X | X |  | X | X |
| *Therapeutic drug monitoring* | | | | | | | | |
| (Nor)clozapine trough level, 12±1 hour after last (evening) dose |  | X^7^ |  |  |  |  |  | X^8^ |
| *Other measurements* | | | | | | | | |
| Albumin | X |  |  |  |  |  |  |  |
| Vitamine B12 | X^9^ |  |  |  |  |  |  |  |
| Folic acid | X^9^ |  |  |  |  |  |  |  |
| Prolactin | X^10^ |  |  |  |  |  |  |  |
| Temperature | X |  |  |  |  |  |  | X |
| EEG |  |  |  |  |  |  |  | X^11^ |
| C-reactive protein |  |  |  |  |  |  |  | X |
| Troponin | X |  | X |  |  |  |  | X |
| Pregnancy test | X^12^ |  |  |  |  |  |  | X^12^ |

^1^ With cardiac anamnesis, age >60 years of use of one or more QTc-prolonging drugs

^2^ At least with significant dose alterations in patients with risk factors (see ^1^)

^3^ Weekly leucocyte- and granulocytecounts for the first 18 weeks, then four-weekly

^4^ In case of a history of liver disease

^5^ FT4 only in case of a deviating thyroid-stimulating hormone level

^6^ HbA_1C_ (combined with a non-fasting glucose) in case a fasting glucose cannot be determined

^7^ Therapeutic drug monitoring can be performed 6 days after the last dose adjustment; many patients reach steady state after three days, so from the fourth day onward a trough level can be measured. Furthermore, therapeutic drug monitoring moments during dose adjustments can be for example after reaching the 100 mg dose, with unexpected severe adverse effects and after reaching the target dose

^8^ For example 14 days after addition or tapering interacting drugs; starting/quiting smoking of excessive caffeine use; in addition, immediate therapeutic drug monitoring in case of dose dependent adverse effects or toxicity (mainly insults, drooling, sedation, hypotension); fever as a consequence of an inflammatory response; check on drug adherence; (danger of) psychotic decompensation

^9^ On indication, in any case with age >65 years

^10^ On indication, in any case with young adults and for example in case of congenital or historic prolactin level deviations

^11^ For example with insults during use of antipsychotics

^12^ In case of uncertainty about a potential pregnancy with women of child-bearing age
