# Supplementary material for: Design and methods of the ‘monitoring outcomes of psychiatric pharmacotherapy’ (MOPHAR) monitoring program – a study protocol
Source: BMC Health Serv Res. 2019 Feb 14;19:125. doi: 10.1186/s12913-019-3951-2 (PMC6376699; doi:10.1186/s12913-019-3951-2)
Supplement: Supplementary file 9 — Table S9. MOPHAR monitoring protocol lithium (DOCX 29 kb) [file 12913_2019_3951_MOESM9_ESM.docx]

**Supplemental table 9. MOPHAR monitoring protocol lithium**

|  | T = 0 | During dose adjustment | At least one measurement between T=3 weeks and T=2 months | T = 3 months | T = 6 months | Every 3 months | Yearly | On indication |
| --- | --- | --- | --- | --- | --- | --- | --- | --- |
| *Anthropometrics* | | | | | | | | |
| Length | X |  |  |  |  |  |  |  |
| Body weight | X |  | X | X |  |  | X |  |
| BMI | X |  | X | X |  |  | X |  |
| Waist circumference | X |  | X | X |  |  | X |  |
| *Cardiovascular measurements* | | | | | | | | |
| Blood pressure (sitting/supine/standing) | X |  |  |  |  |  | X |  |
| Heart rate | X |  |  |  |  |  |  |  |
| Electrocardiogram | X^1^ |  | X^1^ |  |  |  |  | X^2^ |
| *Blood cells* | | | | | | | | |
| Hemoglobin | X |  |  |  |  |  |  |  |
| Hematocrit | X |  |  |  |  |  |  |  |
| Leucocytes | X |  |  | X |  |  |  | X |
| Differential | X |  |  | X |  |  |  | X |
| Thrombocytes | X |  |  |  |  |  |  |  |
| *Electrolytes* | | | | | | | | |
| Sodium | X |  |  | X |  | X |  |  |
| Potassium | X |  |  | X |  | X |  |  |
| Calcium | X |  |  | X |  | X |  |  |
| *Kidney function^3^* | | | | | | | | |
| Creatinin | X |  |  | X |  | X |  |  |
| Estimated Glomerular Filtration Rate (eGFR) | X |  |  | X |  | X |  |  |
| *Liver function* | | | | | | | | |
| Alkaline phosphatase | X |  |  |  |  |  |  |  |
| Alanine transaminase | X |  |  |  |  |  |  |  |
| Gamma-glutamyltransferase | X |  |  |  |  |  |  |  |
| *Schildklierfunctie* | | | | | | | | |
| Thyroid-stimulating hormone + free thyroxine 4 (FT4)^4^ | X |  |  | X |  | X |  |  |
| Thyroperoxidase antibodies |  |  |  |  |  |  |  | X |
| *Blood lipids* | | | | | | | | |
| Triglycerides (fasting) | X |  |  |  |  |  | X |  |
| Cholesterol | X |  |  |  |  |  | X |  |
| Low Density Lipoprotein | X |  |  |  |  |  | X |  |
| High Density Lipoprotein | X |  |  |  |  |  | X |  |
| *Glucose* | | | | | | | | |
| Fasting glucose^5^ | X |  |  |  |  |  | X |  |
| *Therapeutic drug monitoring^6^* | | | | | | | | |
| Lithium level^7^ |  | X^8^ |  |  |  | X^9^ |  |  |
| *Other measurements* | | | | | | | | |
| Albumin | X |  |  | X |  | X |  |  |
| Vitamin B12 | X^10^ |  |  |  |  |  |  |  |
| Folic acid | X^10^ |  |  |  |  |  |  |  |
| Prolactin | X^11^ |  |  |  |  |  |  |  |
| Temperature | X |  |  |  |  |  |  |  |
| Pregnancy test | X^12^ |  |  |  |  |  |  | X^12^ |

^1^ With cardiac anamnesis, age >60 years of use of one or more QTc-prolonging drugs

^2^ At least with significant dose alterations in patients with risk factors (see ^1^)

^3^ Consider measuring a 24-hour urine after consulting a general practitioner/internist in case of a deviating eGFR

^4^ FT4 only in case of a deviating thyroid-stimulating hormone level

^5^ HbA_1C_ (combined with a non-fasting glucose) in case a fasting glucose cannot be determined

^6^ Correction factor for lithium level in case of dosing other than twice a day: 0,9 in case of once a day dosing, 1.2 in case of three times a day dosing

^7^ Blood withdrawal for therapeutic drug monitoring 12±1 hour after the last (evening) dose (12-hours level); <1 hour before the next dose in case of three or four times a day dosing (trough level).

^8^ During dose adjustments: - 3 days after the first dose

- then 5-7 days every dose adjustment and after starting/stopping of interacting drugs

- repeat until two consecutive measurements show constant levels in the therapeutic window, then every 3-6 months (see ^9^).

^9^ After reaching a stable target level; at least every six months

^10^ On indication, in any case with age >65 years

^11^ On indication, in any case with young adults and for example in case of congenital or historic prolactin level deviations

^12^ In case of uncertainty about a potential pregnancy with women of child-bearing age

**Carbamazepine**

|  | T = 0 | Tijdens instelperiodes | Minimaal één meting tussen t = 3 weken en t = 2 maanden | T = 3 maanden | T = 6 maanden | Elke 3 maanden | Jaarlijks | Op indicatie |
| --- | --- | --- | --- | --- | --- | --- | --- | --- |
| *Antropometrie* | | | | | | | | |
| Lengte | X |  |  |  |  |  |  |  |
| Lichaamsgewicht | X |  |  |  |  |  |  |  |
| BMI | X |  |  |  |  |  |  |  |
| Buikomvang | X |  |  |  |  |  |  |  |
| *Cardiovasculaire metingen* | | | | | | | | |
| ECG | X^1^ |  | X^1^ |  |  |  |  | X^2^ |
| *Bloed* | | | | | | | | |
| Hb | X |  | X | X |  |  |  | X |
| Ht | X |  | X | X |  |  |  | X |
| Leukocyten | X |  | X | X |  |  |  | X |
| Differentiatie | X |  | X | X |  |  |  | X |
| Trombocyten | X |  | X | X |  |  |  | X |
| *Elektrolyten* | | | | | | | | |
| Natrium | X |  | X | X |  |  | X |  |
| Kalium | X |  |  |  |  |  |  |  |
| Calcium | X |  |  |  |  |  |  |  |
| *Nierfunctie* | | | | | | | | |
| Creatinine | X |  | X | X |  |  | X |  |
| eGFR | X |  | X | X |  |  | X |  |
| *Leverfunctie* | | | | | | | | |
| AF | X |  |  |  |  |  |  |  |
| ALAT | X |  |  |  |  |  |  |  |
| gammaGT | X |  |  |  |  |  |  |  |
| *Schildklierfunctie* | | | | | | | | |
| TSH + FT4^3^ | X |  |  | X |  |  | X |  |
| *Lipidenspectrum (nuchter)* | | | | | | | | |
| Triglyceriden | X |  |  |  |  |  |  |  |
| Cholesterol | X |  |  |  |  |  |  |  |
| LDL | X |  |  |  |  |  |  |  |
| HDL | X |  |  |  |  |  |  |  |
| *Glucose* | | | | | | | | |
| Nuchter glucose^4^ | X |  |  |  |  |  |  |  |
| *Geneesmiddelspiegel* | | | | | | | | |
| Carbamazepinedal-spiegel |  | X^5^ |  |  |  |  |  | X^6^ |
| *Overige bepalingen* | | | | | | | | |
| Albumine | X |  |  |  |  |  |  |  |
| Foliumzuur | X^7^ |  |  |  |  |  |  |  |
| Vitamine B12 | X^7^ |  |  |  |  |  |  |  |
| Prolactine | X^8^ |  |  |  |  |  |  |  |
| Temperatuur | X |  |  |  |  |  |  |  |
| Zwangerschapstest | X^9^ |  |  |  |  |  |  | X^9^ |

^1^ Bij cardiale anamnese, leeftijd >60 jaar of gebruik van één of meer QTc-verlengende geneesmiddelen.

^2^ In ieder geval bij significante dosiswijzigingen bij patiënten met risicofactoren (zie ^1^)

^3^ FT4 alleen bepalen bij een afwijkende TSH-waarde.

^4^ HbA_1C_ (in combinatie met niet-nuchtere glucosewaarde) als nuchtere glucosewaarde niet kan worden bepaald.

^5^ Tijdens instellen: - vijf-zeven dagen na eerste dosis

- vervolgens vijf-zeven dagen na elke dosisverandering

- 4-6 weken na begin behandeling (i.v.m. mogelijke spiegeldaling door enzyminductie)

De dosis is goed ingesteld als twee achtereenvolgende metingen constante therapeutische bloedspiegels aantonen. Daarna wordt alleen op indicatie een bloedspiegel

bepaald (zie ^6^).

^6^ Na bereiken beoogde én stabiele spiegel; bijvoorbeeld bij bijwerkingen, therapietrouwproblemen, veranderingen van de dosis, enzovoort.

^7^ Op indicatie, in ieder geval bij >65 jaar.

^8^ Op indicatie, in ieder geval bij jong volwassenen en verder bijvoorbeeld bij (congenitale) afwijkingen in het prolactine in de voorgeschiedenis.

^9^ Bij onzekerheid over een eventuele zwangerschap bij vrouwen in de vruchtbare leeftijd.

**Valproïnezuur**

|  | T = 0 | Tijdens instelperiodes | Minimaal één meting tussen t = 3 weken en t = 2 maanden | T = 3 maanden | T = 6 maanden | Elke 3 maanden | Jaarlijks | Op indicatie |
| --- | --- | --- | --- | --- | --- | --- | --- | --- |
| *Antropometrie* | | | | | | | | |
| Lengte | X |  |  |  |  |  |  |  |
| Lichaamsgewicht | X |  | X | X |  |  | X |  |
| BMI | X |  | X | X |  |  | X |  |
| Buikomvang | X |  | X | X |  |  | X |  |
| *Cardiovasculaire metingen* | | | | | | | | |
| Bloeddruk | X |  |  |  |  |  | X |  |
| Hartfrequentie | X |  |  |  |  |  |  |  |
| ECG | X^1^ |  |  |  |  |  |  |  |
| *Bloed* | | | | | | | | |
| Hb | X |  |  | X |  |  |  | X |
| Ht | X |  |  | X |  |  |  | X |
| Leukocyten | X |  |  | X |  |  |  | X |
| Differentiatie | X |  |  | X |  |  |  | X |
| Trombocyten | X |  |  | X |  |  |  | X |
| *Elektrolyten* | | | | | | | | |
| Natrium | X |  |  | X |  |  | X |  |
| Kalium | X |  |  |  |  |  |  |  |
| Calcium | X |  |  |  |  |  |  |  |
| *Nierfunctie* | | | | | | | | |
| Creatinine | X |  |  |  |  |  |  |  |
| eGFR | X |  |  |  |  |  |  |  |
| *Leverfunctie* | | | | | | | | |
| AF | X |  | X | X |  |  | X |  |
| ALAT | X |  | X | X |  |  | X |  |
| gammaGT | X |  | X | X |  |  | X |  |
| *Schildklierfunctie* | | | | | | | | |
| TSH + FT4^2^ | X |  |  |  |  |  |  |  |
| *Lipidenspectrum (nuchter)* | | | | | | | | |
| Triglyceriden | X |  |  |  |  |  | X |  |
| Cholesterol | X |  |  |  |  |  | X |  |
| LDL | X |  |  |  |  |  | X |  |
| HDL | X |  |  |  |  |  | X |  |
| *Glucose* | | | | | | | | |
| Nuchter glucose^3^ | X |  |  |  |  |  | X |  |
| *Geneesmiddelspiegel* | | | | | | | | |
| Valproïnezuurdal-spiegel |  | X^4^ |  |  |  |  |  | X^5^ |
| *Overige bepalingen* | | | | | | | | |
| Albumine | X |  |  |  |  |  |  |  |
| Foliumzuur | X^6^ |  |  |  |  |  |  |  |
| Vitamine B12 | X^6^ |  |  |  |  |  |  |  |
| Prolactine | X^7^ |  |  |  |  |  |  |  |
| Temperatuur | X |  |  |  |  |  |  |  |
| Zwangerschapstest | X^8^ |  |  |  |  |  |  | X^8^ |

^1^ Bij cardiale anamnese, leeftijd >60 jaar of gebruik van één of meer QTc-verlengende geneesmiddelen.

^2^ FT4 alleen bepalen bij een afwijkende TSH-waarde.

^3^ HbA_1C_ (in combinatie met niet-nuchtere glucosewaarde) als nuchtere glucosewaarde niet kan worden bepaald.

^4^ Tijdens instellen: - vijf-zeven dagen na eerste dosis

- vervolgens vijf-zeven dagen na elke dosisverandering

De dosis is goed ingesteld als twee achtereenvolgende metingen constante therapeutische bloedspiegels aantonen. Daarna wordt alleen op indicatie een bloedspiegel

bepaald (zie ^5^).

^5^ Na bereiken beoogde én stabiele spiegel; bijvoorbeeld bij bijwerkingen, therapietrouwproblemen, veranderingen van de dosis, enzovoort.

^6^ Op indicatie, in ieder geval bij >65 jaar.

^7^ Op indicatie, in ieder geval bij jong volwassenen en verder bijvoorbeeld bij (congenitale) afwijkingen in het prolactine in de voorgeschiedenis.

^8^ Bij onzekerheid over een eventuele zwangerschap bij vrouwen in de vruchtbare leeftijd**.**

**Lamotrigine**

|  | T = 0 | Tijdens instelperiodes | Minimaal één meting tussen t = 3 weken en t = 2 maanden | T = 3 maanden | T = 6 maanden | Elke 3 maanden | Jaarlijks | Op indicatie |
| --- | --- | --- | --- | --- | --- | --- | --- | --- |
| *Antropometrie* | | | | | | | | |
| Lengte | X |  |  |  |  |  |  |  |
| Lichaamsgewicht | X |  |  |  |  |  |  |  |
| BMI | X |  |  |  |  |  |  |  |
| Buikomvang | X |  |  |  |  |  |  |  |
| *Cardiovasculaire metingen* | | | | | | | | |
| Bloeddruk | X |  |  |  |  |  |  |  |
| Hartfrequentie | X |  |  |  |  |  |  |  |
| ECG | X^1^ |  |  |  |  |  |  |  |
| *Bloed* | | | | | | | | |
| Hb | X |  |  |  |  |  |  | X |
| Ht | X |  |  |  |  |  |  | X |
| Leukocyten | X |  |  |  |  |  |  | X |
| Differentiatie | X |  |  |  |  |  |  | X |
| Trombocyten | X |  |  |  |  |  |  | X |
| *Elektrolyten* | | | | | | | | |
| Natrium | X |  |  |  |  |  | X |  |
| Kalium | X |  |  |  |  |  |  |  |
| Calcium | X |  |  |  |  |  |  |  |
| *Nierfunctie* | | | | | | | | |
| Creatinine | X |  |  |  |  |  |  |  |
| eGFR | X |  |  |  |  |  |  |  |
| *Leverfunctie* | | | | | | | | |
| AF | X |  | X | X |  |  | X |  |
| ALAT | X |  | X | X |  |  | X |  |
| gammaGT | X |  | X | X |  |  | X |  |
| *Schildklierfunctie* | | | | | | | | |
| TSH + FT4^2^ | X |  |  |  |  |  |  |  |
| *Lipidenspectrum (nuchter)* | | | | | | | | |
| Triglyceriden | X |  |  |  |  |  |  |  |
| Cholesterol | X |  |  |  |  |  |  |  |
| LDL | X |  |  |  |  |  |  |  |
| HDL | X |  |  |  |  |  |  |  |
| *Glucose* | | | | | | | | |
| Nuchter glucose^3^ | X |  |  |  |  |  |  |  |
| *Geneesmiddelspiegel* | | | | | | | | |
| Lamotrigine-dalspiegel |  |  |  |  |  |  |  | X^4^ |
| *Overige bepalingen* | | | | | | | | |
| Albumine | X |  |  |  |  |  |  |  |
| Foliumzuur | X^5^ |  |  |  |  |  |  |  |
| Vitamine B12 | X^5^ |  |  |  |  |  |  |  |
| Prolactine | X^6^ |  |  |  |  |  |  |  |
| Temperatuur | X |  |  |  |  |  |  |  |
| Zwangerschapstest | X^7^ |  |  |  |  |  |  | X^7^ |

^1^ Bij cardiale anamnese, leeftijd >60 jaar of gebruik van één of meer QTc-verlengende geneesmiddelen.

^2^ FT4 alleen bepalen bij een afwijkende TSH-waarde.

^3^ HbA_1C_ (in combinatie met niet-nuchtere glucosewaarde) als nuchtere glucosewaarde niet kan worden bepaald.

^4^ Bijvoorbeeld bij bijwerkingen, therapietrouwproblemen, veranderingen van de dosis, enzovoort. Een spiegel bij stabiele inname en effectiviteit als referentiewaarde kan nuttig zijn.

^5^ Op indicatie, in ieder geval bij >65 jaar.

^6^ Op indicatie, in ieder geval bij jong volwassenen en verder bijvoorbeeld bij (congenitale) afwijkingen in het prolactine in de voorgeschiedenis.

^7^ Bij onzekerheid over een eventuele zwangerschap bij vrouwen in de vruchtbare leeftijd**.**
