# Supplementary material for: Design and methods of the ‘monitoring outcomes of psychiatric pharmacotherapy’ (MOPHAR) monitoring program – a study protocol
Source: BMC Health Serv Res. 2019 Feb 14;19:125. doi: 10.1186/s12913-019-3951-2 (PMC6376699; doi:10.1186/s12913-019-3951-2)
Supplement: Supplementary file 10 — Table S10. MOPHAR monitoring protocol carbamazepine (DOCX 19 kb) [file 12913_2019_3951_MOESM10_ESM.docx]

**Supplemental table 10. MOPHAR monitoring protocol carbamazepine**

|  | T = 0 | During dose adjustment | At least one measurement between T=3 weeks and T=2 months | T = 3 months | T = 6 months | Every 3 months | Yearly | On indication |
| --- | --- | --- | --- | --- | --- | --- | --- | --- |
| *Anthropometrics* | | | | | | | | |
| Length | X |  |  |  |  |  |  |  |
| Body weight | X |  |  |  |  |  |  |  |
| BMI | X |  |  |  |  |  |  |  |
| Waist circumference | X |  |  |  |  |  |  |  |
| *Cardiovascular measurements* | | | | | | | | |
| Electrocardiogram | X^1^ |  | X^1^ |  |  |  |  | X^2^ |
| *Blood cells* | | | | | | | | |
| Hemoglobin | X |  | X | X |  |  |  | X |
| Hematocrit | X |  | X | X |  |  |  | X |
| Leucocytes | X |  | X | X |  |  |  | X |
| Differential | X |  | X | X |  |  |  | X |
| Thrombocytes | X |  | X | X |  |  |  | X |
| *Electrolytes* | | | | | | | | |
| Sodium | X |  | X | X |  |  | X |  |
| Potassium | X |  |  |  |  |  |  |  |
| Calcium | X |  |  |  |  |  |  |  |
| *Kidney function* | | | | | | | | |
| Creatinin | X |  | X | X |  |  | X |  |
| Estimated Glomerular Filtration Rate (eGFR) | X |  | X | X |  |  | X |  |
| *Liver function* | | | | | | | | |
| Alkaline phosphatase | X |  |  |  |  |  |  |  |
| Alanine transaminase | X |  |  |  |  |  |  |  |
| Gamma-glutamyltransferase | X |  |  |  |  |  |  |  |
| *Thyroid function* | | | | | | | | |
| Thyroid-stimulating hormone + free thyroxine 4 (FT4)^3^ | X |  |  | X |  |  | X |  |
| *Blood lipids* | | | | | | | | |
| Triglycerides (fasting) | X |  |  |  |  |  |  |  |
| Cholesterol | X |  |  |  |  |  |  |  |
| Low Density Lipoprotein | X |  |  |  |  |  |  |  |
| High Density Lipoprotein | X |  |  |  |  |  |  |  |
| *Glucose* | | | | | | | | |
| Fasting glucose^4^ | X |  |  |  |  |  |  |  |
| *Therapeutic drug monitoring* | | | | | | | | |
| Carbamazepine trough level |  | X^5^ |  |  |  |  |  | X^6^ |
| *Other measurements* | | | | | | | | |
| Albumin | X |  |  |  |  |  |  |  |
| Vitamin B12 | X^7^ |  |  |  |  |  |  |  |
| Folic acid | X^7^ |  |  |  |  |  |  |  |
| Prolactin | X^8^ |  |  |  |  |  |  |  |
| Temperature | X |  |  |  |  |  |  |  |
| Pregnancy test | X^9^ |  |  |  |  |  |  | X^9^ |

^1^ With cardiac anamnesis, age >60 years of use of one or more QTc-prolonging drugs

^2^ At least with significant dose alterations in patients with risk factors (see ^1^)

^3^ FT4 only in case of a deviating thyroid-stimulating hormone level

^4^ HbA_1C_ (combined with a non-fasting glucose) in case a fasting glucose cannot be determined

^5^ During dose adjustments: - 5-7 days after the first dose

- then 5-7 days after every dose adjustment

- 4-6 weeks after start of treatment (because of possible level decrease because of enzyme induction)

- The dose has been correctly adjusted when two consecutive measurements show constant levels in the therapeutic window; thereafter, therapeutic drug monitoring is only performed on indication (see ^6^)

^6^ After reaching a stable target level; for example with side effects, therapy adherence issues, dose adjustments, etc.

^7^ On indication, in any case with age >65 years

^8^ On indication, in any case with young adults and for example in case of congenital or historic prolactin level deviations

^9^ In case of uncertainty about a potential pregnancy with women of child-bearing age
